# [Corrigendum] Mori Folium water extract alleviates articular cartilage damages and inflammatory responses in monosodium iodoacetate-induced osteoarthritis rats 

**DOI:** 10.3892/mmr.2025.13685

**Published:** 2025-09-16

**Authors:** Jin-Woo Jeong, Hye Hyeon Lee, Jongsik Kim, Eun-Ok Choi, Hyun Hwang-Bo, Hong Jae Kim, Min Young Kim, Kyu Im Ahn, Gi-Young Kim, Ki Won Lee, Ki Young Kim, Sung Goo Kim, Su Hyun Hong, Cheol Park, Hee-Jae Cha, Yung Hyun Choi

Mol Med Rep 16: 3841–3848, 2017; DOI: 10.3892/mmr.2017.7075

Subsequently to the publication of the above paper, an interested reader drew to the authors’ attention that, concerning the histological data shown in Fig. 2A on p. 3844, the “Control / Toluidin Blue” data panel contained a section of data that was strikingly similar to data that had appeared previously in Fig. 2 in another article written by some of the same authors in *EXCLI Journal.* In addition, the “MF / CTX–II” data panel was similarly strikingly similar to another data panel in Fig. 2 of the same article appearing in *EXCLI Journal.* Finally, it was also noted that the “MF” panel in Fig. 3 contained an overlapping section with the “MIA+MF / COX-2” data panel in Fig. 6.

After having re-examined their original data, the authors realize that Figs. 2, 3 and 4 of the above paper had contained errors in terms of their assembly. The revised versions of Figs. 2, 3 and 4, now showing different histological images derived from two of the parallel experiments with a different magnification (scaled to 100 μm; the original images were scaled to 200 μm), are shown on the next two pages. Note that these errors did not adversely affect either the results or the overall conclusions reported in this study. All the authors agree with the publication of this corrigendum, and are grateful to the Editor of *Molecular Medicine Reports* for allowing them the opportunity to publish this. They also wish to apologize to the readership of the Journal for any inconvenience caused.

## Figures and Tables

**Figure 2.**
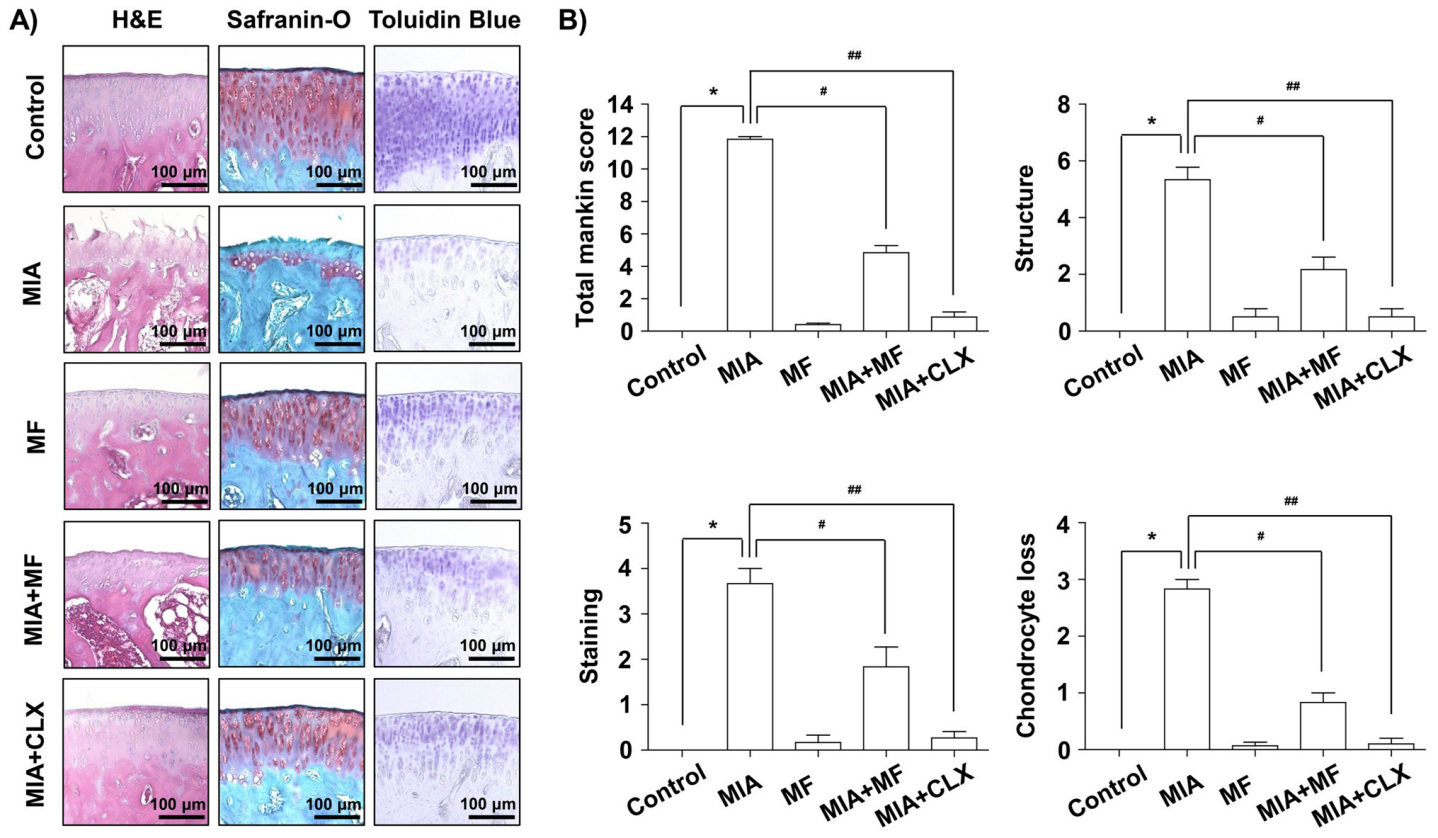
Histological evaluation of joints activity after administration with MF in MIA-induced OA rats. The rats were injected with 3 mg MIA in the right knee, and then CLX was administered orally once a day for 3 weeks after the MIA injection. (A) Knee joints of the OA rats were stained with H&E, Safranin-O and toluidine blue. (B) The joint lesions were graded on a scale of 0-13 using the modified Mankin scoring system, giving a combined score for cartilage structure, cellular abnormalities and matrix staining. Data are expressed as the mean ± standard deviation (n=8). *P<0.05, **P<0.01. H&E, hematoxylin and eosin; MIA, monosodium iodoacetate; MF, Mori folium; CLX, celecoxib.

**Figure 3.**
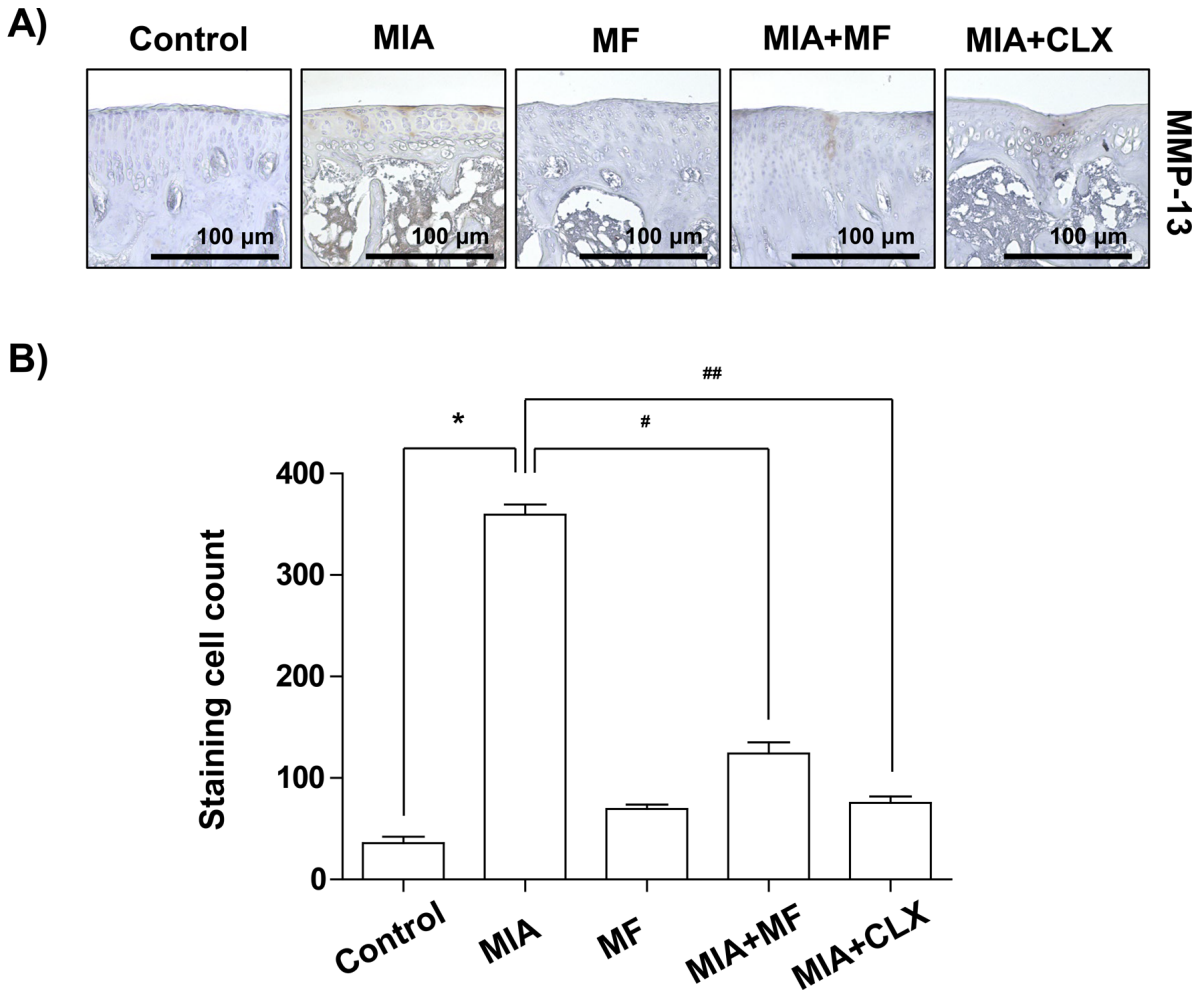
Effects of MF on the expression of MMP-13 in MIA-induced OA rats. (A) Immunohistochemical staining was used to identify the expression of MMP-13 in the articular cartilage. (B) Stained cells wren counted and data are expressed as mean ± standard deviation (n=8). *P<0.05, **P<0.01. MIA, monosodium iodoacetate; MF, Mori folium; CLX, celecoxib; MMP, matrix metalloproteinase

**Figure 4.**
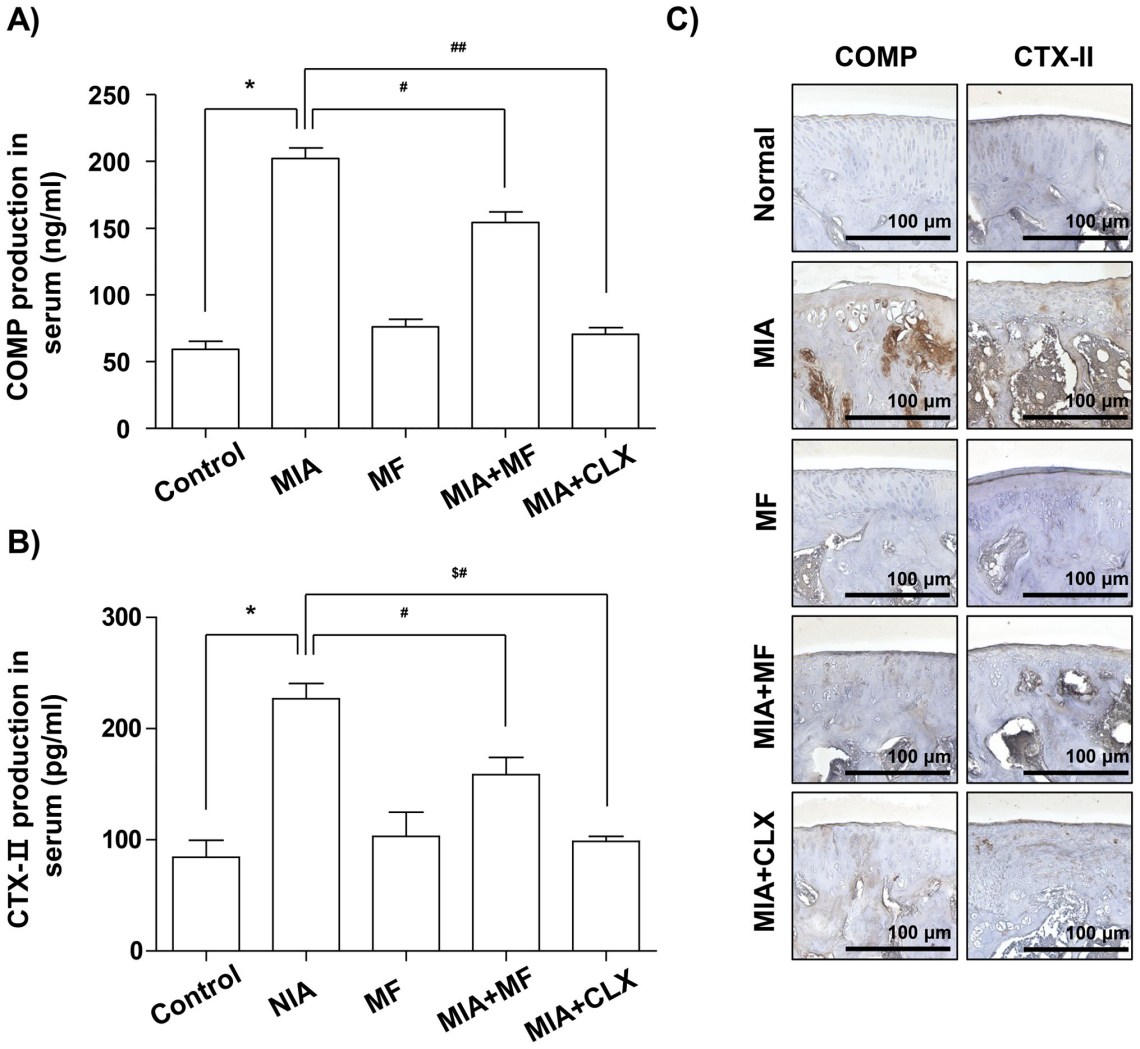
Effects of MF on the production and expression of COMP and CTXII in MIA-induced OA rats. (A) COMP and (B) CTXII production was measured in the serum of MIA-induced OA rats using commercial ELISA kits. (C) Immunohistochemical staining was used to identify the expression of COMP and CTX–II in the articular cartilage. Data are expressed as the mean ± standard deviation (n=8). *P<0.05, **P<0.01. MIA, monosodium iodoacetate; MF, Mori folium; CLX, celecoxib; COMP, cartilage oligomeric matrix protein; CTX–II, C-telopeptide of type II collagen..

